# The Regulatory Role of the Human Mediodorsal Thalamus

**DOI:** 10.1016/j.tics.2018.08.006

**Published:** 2018-11

**Authors:** Giulio Pergola, Lola Danet, Anne-Lise Pitel, Giovanni A. Carlesimo, Shailendra Segobin, Jérémie Pariente, Boris Suchan, Anna S. Mitchell, Emmanuel J. Barbeau

**Affiliations:** 1 Department of Basic Medical Sciences, Neuroscience and Sense Organs, University of Bari Aldo Moro, Bari 70124, Italy; 2 Toulouse NeuroImaging Center, Université de Toulouse, Inserm, UPS 31024, France; 3 CHU Toulouse Purpan, Neurology Department, Toulouse 31059, France; 4 Normandie University, UNICAEN, PSL Research University, EPHE, INSERM, U1077, CHU de Caen, Neuropsychologie et Imagerie de la Mémoire Humaine, 14000 Caen, France; 5 Department of Systems Medicine, Tor Vergata University and S. Lucia Foundation, Rome, Italy; 6 Clinical Neuropsychology, Ruhr University Bochum, Universitätsstrasse 150, 44801 Bochum, Germany; 7 Department of Experimental Psychology, University of Oxford, The Tinsley Building, Mansfield Road, Oxford OX1 3SR, UK; 8 Centre de recherche Cerveau et Cognition, UMR5549, Université de Toulouse – CNRS, Toulouse 31000, France; 9 Equivalent contribution as last authors

**Keywords:** mediodorsal thalamus, memory, neuroimaging, persistent activity, prefrontal cortex, temporal extension

## Abstract

The function of the human mediodorsal thalamic nucleus (MD) has so far eluded a clear definition in terms of specific cognitive processes and tasks. Although it was at first proposed to play a role in long-term memory, a set of recent studies in animals and humans has revealed a more complex, and broader, role in several cognitive functions. The MD seems to play a multifaceted role in higher cognitive functions together with the prefrontal cortex and other cortical and subcortical brain areas. Specifically, we propose that the MD is involved in the regulation of cortical networks especially when the maintenance and temporal extension of persistent activity patterns in the frontal lobe areas are required.

## Highlights

The mediodorsal thalamic nucleus is involved in the cognitive deficits observed in several neurological and psychiatric disorders.

The long-standing belief in a role of the mediodorsal thalamic nucleus mainly in long-term memory is now being reconsidered. Recent studies emphasize its function in many cognitive tasks related to the prefrontal cortex.

The mediodorsal thalamic nucleus is required for the rapid and accurate performance of cognitive tasks and temporally extends the efficiency of cortical networks involving the prefrontal cortex.

We propose that the common ground of multiple lines of evidence from human studies points to a role of the mediodorsal thalamic nucleus in regulating prefrontal activity patterns.

These hypotheses can be tested by developing specific neuropsychological tasks, parceling the thalamus with high-resolution MRI, and using intracranial recordings in humans.

*Nevertheless, Euryclea, take his bed outside the bed chamber that he himself built.*

Odyssey, Book XXIII, verses 177–178. Butler translation.

## The Mediodorsal Nucleus: A Reappraisal

When faced with the prospect of welcoming a stranger as her long-missing husband, and unable to recognize him, Penelope resorted to her ‘thalamus’ with the words reported above – the bed had roots in the foundation of the house and could not be moved, a detail her husband would certainly know. Initially entering the scene as something instrumental to recognition, the **thalamus** (see Glossary) in the Odyssey becomes the center of the scene in the last two books.

Likewise, in neuroscience, the investigation of the **mediodorsal thalamic nucleus** (MD) is gaining momentum. Until recently, the function of the MD has been mapped onto specific cognitive domains, such as memory or executive function. An influential model on a role of the MD in recognition memory for instance suggested it might play a role in familiarity [1]. However, abundant evidence indicates that this view is limited and that the role of the MD in human cognition must be reconsidered. For example, clinicians have known for a long time that the MD and its brain networks are involved in several neurological and psychiatric conditions in which the cognitive deficits are not restricted to memory functions [2]. Neuroimaging and neurophysiology studies of the human MD *in vivo* further support this view change.

This review evaluates the latest evidence in humans and aims at formulating hypotheses to elucidate the cognitive functions of the human MD in future studies. We argue that the MD is involved in regulating activity patterns in the frontal lobe that are key to perform cognitive functions characterized by persistent thalamocortical interactions for long delays, in the face of interference, and during multitasking. Disruptions to this thalamofrontal communication may in turn underlie cognitive deficits in several neurological and psychiatric conditions and represent a possible therapeutic target.

## Beyond Recognition Memory, from Rodents to Humans

**Figure 1:**
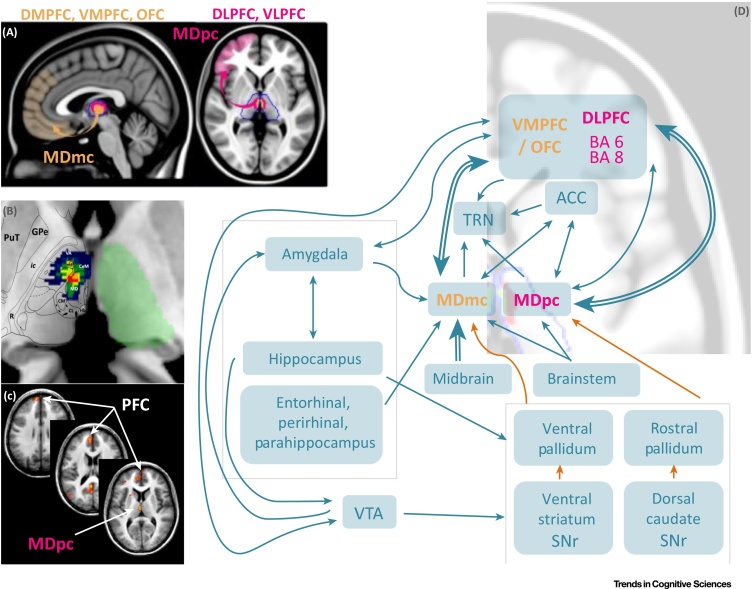
Connectivity of the MD in Primates. (A) On the left side: connections between the magnocellular MD and the ventromedial and orbitofrontal areas of the PFC. On the right side: connections between the parvocellular MD and the lateral PFC. (B) Overlap of the medial lesions involving the MD of 12 stroke patients (modified from [36]). (C) fMRI activation maps involving the MD and the PFC during episodic retrieval in healthy subjects (modified from [73]). (D) Diagram showing the structural connectivity of the magnocellular MD and the parvocellular MD. Blue arrows correspond to excitatory connections. Orange arrows correspond to inhibitory connections. Abbreviations: ACC, anterior cingulate cortex; CeM, central medial thalamic nucleus; CL, central lateral thalamic nucleus; CM, centromedian thalamic nucleus; DLPFC, dorsolateral prefrontal cortex; DMPFC, dorsomedial prefrontal cortex; GPe, external globus pallidus; Hb, habenula; Ic, internal capsule; MD, mediodorsal thalamic nucleus; MDmc, magnocellular subdivision of the mediodorsal thalamic nucleus; MDpc, parvocellular subdivision of the mediodorsal thalamic nucleus; mtt, mammillothalamic tract; OFC, orbitofrontal cortex; PFC, prefrontal cortex; PuT, putamen; R, thalamic reticular nucleus; SNr, substantia nigra pars reticulata; TRN, thalamic reticular nucleus; VA, ventral–anterior thalamic nucleus; VLPFC, ventrolateral prefrontal cortex; VMPFC, ventromedial prefrontal cortex; VTA, ventral tegmental area.

Animal models emphasizing the role of the MD in recognition memory and familiarity were based on its monosynaptic input from the perirhinal cortex in primates [1] (while this pathway is only weak in rodents [3]). Earlier pioneering work in non-human primates, however, had demonstrated the influence of MD–**prefrontal cortex** (PFC) interactions on MD activity across delays 4, 5. Recent evidence in rodents 6, 7, 8 and in monkeys 9, 10 indicates that the MD influences multiple cognitive abilities via its interactions with areas of the frontal lobe, such as the PFC and anterior cingulate cortex, to which the MD is reciprocally connected in rodents as well as in primates 11, 12, 13 (Figure 1). Individual MD neurons from different subdivisions of the MD exhibit a considerable degree of divergence in their projections, that is, each rodent MD neuron projects to several different PFC subdivisions [14]. Similarly, in monkeys and humans, MD efferences diverge and make contact with multiple PFC areas 15, 16. The MD is thus interacting with many frontal areas simultaneously, which in turn have intrinsic connections among multiple cortical layers [17]. MD neurons may directly facilitate corticocortical communication via trans-thalamic pathways [18], and it would be important to further support this hypothesis with neurophysiological measurements [19].

What has been convincingly demonstrated is that persistent PFC activity patterns depend on MD inputs and on recurrent excitation of thalamofrontal circuits 6, 20. Hence the MD may have a role, not limited to **long-term memory** (LTM), in sustaining delay-related activity in the PFC [21]. Interestingly, while the initial maintenance may be sustained by the PFC alone, its interactions with the MD could extend this activity pattern from several seconds to several minutes and possibly beyond [21]. The temporal regulation of the mutual interdependence of MD and PFC activity, for example, the rapid adjustment of the phase and frequency of cortical oscillations 7, 9, 13, is an idea also common to other views 18, 22, 23. Thus, the MD when actively engaging with the PFC might support synaptic reverberations in recurrent thalamofrontal loops that promote persistent activity across several cortical regions necessary for efficient cognitive functioning. In other words, the influence of the MD on the cortex may allow for reflections, decisions, and actions relevant to the current task demands to extend over a window of time that is contextually relevant and unfolds at temporal scales distinct in different mammalian species.

These ideas, mainly developed through experiments in rodents and monkeys, are plausible in humans too, and these advances call for timely translations into the human field. We are well aware that there is still much to learn in terms of establishing clear homologies between animal models and humans. For example, the primate MD includes an intrinsic population of interneurons releasing GABA that has not been identified in rodents [24]. In primates, the MD is also rich in dopamine receptors receiving their input from multiple independent pathways 25, 26, making it part of well-studied brain networks involved in saliency detection [27]. Further, the MD is part of a primate-specific network linking the amygdala with the thalamic reticular nucleus [28], one of the main sources of GABA within the thalamus. Importantly, animals are typically overtrained on the tasks they perform, whereas it has been argued that novel, complex tasks, not easily solved based on procedures, expertise, or overlearned knowledge, should particularly tap MD–PFC interactions [13]. Yet we contend that general principles learned from experimental animal models are not undermined by these differences, because of the generally similar connectivity patterns of the MD with the PFC across species. If anything, the primate-specific MD features make it more central in brain networks relevant to cognition (Figure 1).

Here we propose that such species-specific adaptations, rather than establishing different functions in the primate context compared to rodents, reflect the phylogenetic adaptation of the interactions between the MD and the PFC in the context of a generally different organization of the primate brain. For example, while saliency detection and persistent PFC activity across delays are affected by MD dysfunction in rodents, the neurochemical basis is likely different to that in primates, since the latter takes advantage of dopamine inputs to the MD. Specifically, we argue that the human MD is in a unique position to participate in the activity of multiple brain networks that exceed the definition of a single cognitive domain – a notion that, in rodents and non-human primates, is supported by multiple lines of evidence 7, 13, 29. To this aim, we review the available evidence in human studies from the perspective of clinical, neuroimaging, and neurophysiological studies that highlight the importance of MD–PFC interactions.

## Thalamic Stroke Studies

In humans, the MD has initially been associated with LTM based on its assumed involvement in Korsakoff’s syndrome (KS) 30, 31 (Box 1 reports a historical perspective of the function of the MD in memory). Thalamic stroke studies have also historically played an important role in shedding light on the function of individual thalamic nuclei. In particular, ischemia in the paramedian or tuberothalamic artery or hemorrhage causes MD damage 32, 33. Because of the small size of thalamic nuclei (Box 2), vascular lesions are necessarily unselective and involve multiple nuclei, which also play a role in the ensuing cognitive deficits. For this reason, it is crucial to quantitatively estimate the volume loss separately for different nuclei, an approach that became viable only recently thanks to advances in neuroimaging techniques but that has been too rarely undertaken so far 34, 35, 36. Lesion quantification is challenging especially for lesions proximal to the third ventricle: these infarcts tend to merge with the ventricle, or the ventricle itself undergoes progressive enlargement associated with tissue shrinkage, hindering volume measurements in the medial nuclei (see Outstanding Questions).

Box 1 **Familiarity or Recollection? An Historical Overview**

The interest on thalamic nuclei as higher-order cognition substrates enjoyed a widespread increase after Aggleton and Brown’s review [1]. The authors described the substrates of the two processes underpinning recognition memory: recollection, the ability to retrieve part of the experience associated with a stimulus, and familiarity, the mere feeling that a stimulus has been experienced. They suggested that the circuit linking the hippocampus with the anterior thalamic nuclei (ATN) along with the mammillary bodies and the mammillothalamic tract (MTT) supported recollection. They also proposed that a second independent circuit involved the perirhinal cortex and the MD processed familiarity due to their direct connections. Although a critical role for the ATN in recollection remains undisputed, the role of the MD in familiarity is still contested. Indeed, studies have typically reported impaired recollection with relatively preserved familiarity following MD damage 38, 99, 100, 101. Aggleton and colleagues [102] thus revised their model integrating the specific connectivity pattern of each thalamic nucleus. The multieffect multinuclei model described a functional continuum, rather than a dissociation, between the MTT/ATN and MD via the midline and intralaminar nuclei. In particular, they proposed recollection to be impaired following MD damage because of the dense connections between this nucleus and prefrontal areas, hence switching the role of this nucleus away from its relation with the medial temporal lobes. A distinction was also drawn between the parvocellular MD, which may be involved in recollection (due to their dense connectivity with the PFC), and the magnocellular MD, whose role remains more elusive [102]. Subsequent studies, including those with more refined imaging approaches to localize lesions, appeared in agreement with these proposals 36, 37, 51, leaving the purported role of the MD in familiarity unsubstantiated with the exception of a single case study [103] and fMRI studies 74, 75, 76. Recently, a single case study assessed the impact of MD damage sustained at birth on the Forced Choice Corresponding test, which requires subjects to recognize stimuli among similar foils. Performance on this task is thought to critically depend on the perirhinal cortex, and, by extension, on the MD as a trans-thalamic relay to prefrontal areas. The patient was indeed impaired on trials filled with visual interference, which indicates that the MD may be involved in some visually demanding recognition memory tasks, without necessarily mapping on the classical familiarity/recollection distinction [42].

Alt-text: Box 1

Box 2 **Trends in Magnetic Resonance Imaging of Thalamic Nuclei**

From an MRI perspective, measurements need precision (the minimum possible error in estimating the signal in a voxel) and accuracy (free of artifacts, well-localized signals). Owing to their small size [104] and similarity in terms of relaxation times and/or proton densities, segmenting thalamic nuclei is particularly challenging.

**Structural Scans**

Manual segmentation of thalamic nuclei depends on good contrast between nuclei of interest and neighboring regions. The optimum choice is to use higher field strength (e.g., 7 T), offering higher contrast and signal-to-noise ratio (SNR). Acquisitions are typically study and nuclei specific. Prior reports employed inversion recovery-turbo spin echo [105] for imaging the dorsal thalamus, susceptibility-weighted imaging [106] for its ventral intermediate aspects, and MPRAGE sequences that nullify white matter that separates several nuclei [107]. A trade-off is needed between SNR and acquisition times. Shorter acquisition times help minimize movement artifacts due to head motion or cerebrovascular pulsation [105], while longer acquisition times allow for higher-resolution images, decreasing the mixture of tissues in a voxel. Typically, 7-T MRI sequences with discernable nuclei had acquisition times of 7–15 min for image resolutions varying from 0.67 mm isotropic to 0.375 × 0.375 × 1 mm^3^ [107].

Currently, with most data collected at lower field strength (3 T), automatic segmentation is often preferred. It can be achieved through a histological atlas [108] and requires normalization of the MRI into atlas-standardized space. Its accuracy is limited to the resolution of both, MRI and atlas, and does not account for intersubject variability of shape and volume for each nucleus, especially in patients [109].

**Structural Connectivity**

Automatic segmentation can also employ diffusion imaging and state-of-the-art tractography algorithms. The connectivity strength in each thalamic voxel is evaluated with respect to *a priori*-defined regions 110, 111, 112 or every voxel in the brain [113] and then clustered together according to connectivity-based feature similarities to segment the thalamus [114].

**Functional Connectivity**

Resting-state fMRI studies have segmented the thalamus based on functional connectivity patterns with cortical areas (e.g., independent component analysis 115, 116 or normalized spectral clustering [117]). Thalamic parcels typically do not have a one-to-one mapping to cortical regions and are shared among functional networks [113].

Overall, despite their lower resolution, connectivity-based segmentations are reliable to discriminate thalamic regions since measurements are taken from a global scale and not voxel level 118, 119, 120. However, the inherently low resolution prevents isolation of specific nuclei and rather leads to grouping them (dorsomedian, ventrolateral, anterior, and posterior) [70].

Alt-text: Box 2

Despite these methodological limitations, two of the largest group studies of ischemic focal thalamic lesions to date agreed on a mild-to-moderate LTM impairment of chronic patients with MD lesions, which could not be explained by concurrent lesions of the hippocampal–thalamic axis 36, 37. Short-term memory, including **working memory** (WM), deficits are not consistently reported in group studies, with few positive findings 36, 38. This lack of evidence about WM deficits in stroke patients with focal MD lesions is important because reports based on some animal models emphasized a role of the MD in WM 39, 40, 41. The poor consensus between clinical reports might also be related to sparse human evidence following bilateral lesions. These bilateral lesions likely cause more severe impairment than unilateral ones, but occur more rarely. Studies in patients with bilateral lesions may thus reveal deficits otherwise too mild to be clearly identified in patients with unilateral lesions 33, 42. Other methodological issues could also contribute to the conflicting evidence: for example, the use of span tasks reflects more directly passive storage abilities than other components of WM (i.e., manipulation, interference control, or updating) [43]. Based on further findings reviewed below, these key skills may be particularly affected after MD damage.

**Executive functions, attention control**, prospective memory, arousal, motivation, language, and behavioral deficits are also often reported in the acute phase of focal MD lesions 44, 45, but in general LTM deficits outlast them [33]. At the chronic phase (>3 months after lesion onset), most patients have a poorly defined complaint of being less efficient or having slight memory problems. The functional outcome of these patients is largely unknown (see Outstanding Questions). On this basis, there is no agreement on a clinically relevant chronic outcome of MD damage [46], except perhaps for a mild LTM impairment.

Overall, the loss of cognitive functions after MD damage in humans appears poorly defined. It is possible that damage to the MD is neither necessary nor sufficient to instantiate chronic deficits in other cognitive domains than LTM. Alternatively, and this is the option we explore here, the standard tests used to reveal non-mnemonic deficits may be insufficiently sensitive for elucidating the kind of impairments that occur in humans after MD lesions. For example, many patients with frontal lobe damage show little deficits on standard tests, yet are severely impaired in their professional and family lives [47]. In the 1990s, this lack of clear impairment in laboratory tests was rectified with the development of novel tests (i.e., requiring performance of several tasks within a limited amount of time using a strategy that the patients have to develop themselves; Box 3). We propose that, just as was the case with frontal lobe dysfunctions back in the 1990s, deficits going beyond LTM impairments have likely been underestimated because of the paucity of cases, tests employed, and confounding effects of lesion laterality along with poor measurements of lesions.

Box 3 **Cognitive Tasks to Assess MD Functions in Humans**

We suggest, in this review, that most standard neuropsychological tests are relatively insensitive to identifying human MD functioning. The intrinsic connectivity of the PFC clearly supports many cognitive functions on its own and therefore human MD lesions may cause only moderate or nonspecific impairments in PFC executive functions when assessed with standard neuropsychological tests. In this context, special tests are needed to account for the specific contribution of the MD to cortical PFC functioning. Given that MD neurons are interconnected to many PFC regions, their role is probably more evident in tasks with multifaceted cognitive demands.

We propose the use of tests that meet some of the functional characteristics of the MD outlined in this review. For example, manipulation of internal representations, including memory, predictive coding, goal, rules, susceptible to degradation due to cognitive load, adaptive decision making, multitasking, interference, or long delays, up to several minutes (e.g., >5–30 min) requires strong interactions among PFC regions as well as temporal and spatial extensions, and hence may identify an MD contribution.

One possibility is to use known neuropsychological tests that already meet these criteria. For example, the six elements test [121] requires managing several tasks following complex rules for up to 10 min. Similarly, the so-called self-ordered-pointing task [122] focuses on self-organized arrangements and performance of responses as well as continuous monitoring of one’s performance. A third example is a special span task developed to evaluate the relationships between episodic memory and WM (i.e., the episodic buffer). It requires the integration and memorization of letters and spatial locations to assess the functions of binding and maintenance of multimodal information while manipulating duration of storage and memory load [123]. An alternative is to develop specific, hypothesis-driven, new neuropsychological tasks. For instance, episodic memory studies featuring multiple delays or sources of interference found increased degradation of the memory trace in patients with MD lesions relative to healthy controls 42, 51. Thus, varying temporal parameters, and/or switching demands or manipulating cognitive load will, in our view, provide insights into the influence of the MD on PFC functioning. Lastly, adapting animal tasks shown to identify PFC functioning after selective MD lesions 10, 124 will be insightful. Importantly, we argue that the field will benefit most from hypothesis-driven tasks accompanied by accurate neuroimaging of the consequences of thalamic changes.

Alt-text: Box 3

Further, we suggest that the development of *ad hoc* neuropsychological tests to investigate the MD may reveal novel insight especially regarding the temporal parameters affecting task performance, such as reaction times and response deadlines. Animal studies revealed a function of the MD in persistent PFC activity which would be consistent with the impairments shown by patients with frontal lobe damage in self-paced executive tests. However, the available evidence on the temporal parameters in patients with MD lesion is sparse and the neuropsychology findings on this topic are contradictory (e.g., [48] reported no effects; [37] reported increased reaction time in patients relative to healthy controls). This aspect of MD function may even affect the attribution of deficits to underlying processes based on their timescale. For example, familiarity-based responses (Box 1) may be faster than recollection-based ones 49, 50. Notably, if the different temporal regulation of cognitive operations is collinear to the operations at hand, then what appears to be a qualitative difference between separate cognitive substrates may in part be related to an underlying role of the MD in supporting temporal aspects of the performance. As another example, on a longer timescale (24 h after learning), patients with thalamic ischemia encompassing the MD and other thalamic nuclei show accelerated forgetting [51]. This is case in point that LTM deficits of patients with thalamic lesions may be partly explained by a role of the MD for example temporally extending – higher cognitive functions subserved by frontal lobe areas.

## Clinical Conditions with Gradual Development of MD Dysfunction

Among a number of pathologies that have been associated with the MD, alcohol use disorder (AUD; see Box 4), KS, and schizophrenia (SCZ) are of particular interest. The changes associated with these disorders usually evolve slowly over time. Thus, this evidence complements the lesion studies as it reflects gradual rather than abrupt loss of function. This characteristic yields the potential to follow the relationship between neuroimaging readouts and cognitive/behavioral outcomes over time.

Box 4 **The Thalamus in Alcohol Use Disorder, Korsakoff’s Syndrome, and Diet**

Historically, the link between the thalamus and cognition originates from studies on KS, primarily with excessive alcohol consumption [125]. Alcoholism mainly affects the fronto-cerebellar (including the MD) and Papez circuits [126], which share the thalamus as a key node. Recent neuroimaging investigations have confirmed neuropathological studies, detailing alterations to thalamic volume and structural connectivity in AUD patients even without KS 53, 127. KS onsets when excessive alcohol consumption is combined with thiamine (vitamin B1) deficiency (TD), and is characterized by a profound, global amnesia. AUD patients are at special risk for TD notably because of altered thiamine metabolism. It remains unclear whether the marked changes to the brain observed in KS occur as a result of the neurotoxic effects of alcohol, or sustained TD, or a combination of both [128]. The brain and neuropsychological recovery observed after abstinence in patients with AUD without amnesia 54, 129 suggests that alcoholism alone may not systematically lead to persistent brain dysfunction. Global amnesia in KS remains even after cessation of alcohol use. Thus, severe and persistent damage to the thalamus observed in KS likely results from TD rather than alcohol *per se*, as also suggested by the description of KS without a history of AUD but with systematic nutritional deficits (e.g., bariatric surgery, anorexia).

Animal models have been essential to determine the respective contributions of excessive alcohol consumption and TD to the development of alcohol-related brain damage [130]. These causative studies in rodents have established that chronic and heavy alcohol intake is not mandatory to mimic the specific thalamic alterations observed in KS [131], but alcohol may potentiate the effects of TD [132]. In AUD patients, altered thiamine metabolism was solely predictive of episodic memory impairments [133] and lower levels of circulating thiamine diphosphate selectively correlated with poorer episodic memory performance [134].

The thalamus is not homogeneously affected by TD. The medial and midline thalamic nuclei, and the anterior thalamic nuclei are especially damaged in KS compared with AUD patients without amnesia [53], and in pyrithiamine-induced TD rats [130], reinforcing the idea that these nuclei and their connections play a crucial role in memory [135]. By contrast, the MD is damaged in AUD patients, but not especially in KS patients, or in animal models of KS. Thus, the fronto-cerebellar circuit, including the MD, may not be especially vulnerable to TD, but rather to other comorbid alcohol-related brain dysfunction [133].

Alt-text: Box 4

The MD and other regions of the medial diencephalon have been proposed to underlie the pathophysiology of KS [52]. *In vivo* neuroimaging studies have shown shrinkage of anterior and medial thalamic nuclei in patients with AUD and KS ([53]; see Box 4). Consistently, AUD, KS, as well as SCZ are characterized by deficits of attention, WM, and executive function 54, 55. However, joint evaluations of the neurologic and psychiatric literature aimed to inform the investigation of MD functions have been rare so far 13, 56.

In SCZ, post-mortem studies revealed gray matter reduction and neuronal loss in the thalamus of patients, although this evidence is unspecific with respect to the MD 57, 58. Neuroimaging studies support the idea of thalamic neuropathology in patients with SCZ [59], with longitudinal gray matter changes in the thalamus associated with cognition measures [60]. Volume loss appears nonhomogeneous across thalamic nuclei and shows greater effects in the medial aspects of the thalamus [61]. Unfortunately, very few neuroimaging studies of patients have performed thalamic parcellation, and neuroimaging quantitative assessments of the medial thalamus may be confounded by enlargements of the third ventricle. Nevertheless, recent evidence identified MD gray matter estimates as the top-ranking thalamic feature discriminating patients with SCZ from controls using multivariate statistical analyses [62]. Likewise, longitudinal changes in thalamic gray matter in patients with SCZ appear localized especially in the midline thalamic nuclei and MD [63].

The discrepancy between the post-mortem and structural neuroimaging evidence invites caution, as motion artifacts, effects of medication on brain perfusion, and metabolic state may bias gray matter estimates [64]. In addition, it is unclear whether alterations in the MD are a cause or a consequence of the disease, as multiple studies failed to associate decreased thalamic gray matter with genetic risk for SCZ [61]. Therefore, MD damage may be a consequence of the illness course, potentially confounded by medication or symptom severity progression [58].

Relevant to this review, functional imaging tasks reveal differences between patients with SCZ and healthy controls that are not confined to LTM, although episodic memory alterations are well supported [65]. For instance, medial thalamic regions are hypoactive in SCZ during attention and WM tasks [61]. In addition, the functional connectivity between the thalamus and the PFC is decreased in patients with SCZ, and in their siblings, both during resting state 66, 67, 68 and during attention control [69]. This thalamo–PFC functional connectivity alteration at resting state was also found in individuals at risk or in early disease stages and was associated with verbal learning and memory performance in patients with psychosis 70, 71. The thalamic region disconnected from the PFC was located in a medial thalamic territory compatible with the localization of the MD 69, 70. In summary, although only few studies considered the heterogeneity of thalamic nuclei, the MD and its PFC networks appear dysfunctional in patients with SCZ and in their relatives, associating functional changes in MD activity and connectivity with the genetic component of SCZ, with illness course, and importantly, with effects on cognition wider than LTM. It will be relevant for future studies to characterize the cognitive and clinical correlates of such alterations, as the link of MD dysfunction with longitudinal aspects of the illness highlights that such MD–PFC interactions may represent a therapeutic target (see Outstanding Questions) 60, 63, 71, 72.

## Neuroimaging and Neurophysiology Recordings of the MD

Functional investigations of the activity of the MD in relation to PFC networks are key to address its elusive role in cognition, but until recently, few reports have focused on the function of thalamic nuclei in healthy individuals, partly because of the challenges of thalamic parcellation (Box 2). In agreement with lesion evidence, two decades of **fMRI** studies – some of which are discussed below – have found that the MD is activated during episodic memory retrieval. Three points about this literature are of interest to this review. First, fMRI findings implicating the MD in LTM are outnumbered by studies assessing other cognitive functions, such as emotion processing, reward evaluation, saliency detection, attention control, and executive function. Although the signal associated with different MD subdivisions or the close-by intralaminar nuclei may hinder nuclei-specific inferences in fMRI studies (Box 2), a selective role of the human MD in LTM seems outweighed by its putative involvement in other cognitive functions. Second, although the MD is activated during recall, interindividual variability in MD blood oxygen level-dependent signal is not associated with recall accuracy [73]. Third, MD activity seems unspecific for recall, as tasks emphasizing familiarity more than recollection elicit similar or greater activity during familiarity trials 74, 75, 76. These studies suggest that the modalities of the experimental procedure, that is, an instruction that emphasizes either recall or familiarity and thus directs the attention of the participants towards a target, may affect MD activity [56].

If the MD is not directly related to a specific memory component, it may rather subserve a general role in goal-directed behavior beyond LTM, that is, in persistent activity underlying different types of learning 77, 78, 79, 80, 81, 82. Several studies have further suggested that the MD may process the allocation of attention and the interaction between attention and learning processes in a task-relevant way 29, 83. In this light, the signal detected in the MD during episodic memory performance may represent the temporal activation of a recurrent fronto-thalamic loop that is being maintained during information processing in cortical networks. It follows that the human MD may be activated when tasks require the maintenance of persistent neural activity in areas of the frontal lobe and beyond.

Intracranial neurophysiological recordings in the human MD provide critical insight for this proposal. A single-patient study found that stimulus-linked oscillatory synchrony between the MD and frontal surface electrodes was enhanced for successful recognition memory retrieval compared with successful correct rejections of new items [84]. A Granger causality analysis suggested that the direction of this connectivity was thalamocortical, hence supporting the idea that the MD would enhance prefrontal activity during LTM retrieval. Another intracranial neurophysiological study assessing both encoding and retrieval found that MD prestimulus activity during encoding predicted memory success in an incidental encoding task [85]. MD synchrony with frontal theta waves predicted successful encoding, consistent with fMRI and lesion evidence on the involvement of the MD during encoding 56, 73. In addition, even MD resting state activity unrelated to the task was associated with successful memory formation across participants. These results suggest that MD–PFC interactions are associated with an overall cognitive disposition to successful memory formation, even when that is not the task goal – which is surprising in light of the fMRI literature supporting a role of the MD in goal-related behavior, and hence requires special consideration in models of human MD function. The findings from a third study further eroded the concept that the MD is primarily involved in LTM [86]. A group of patients with epilepsy undergoing intracranial electrode surgery for deep brain stimulation performed a complex executive function task tapping into attention, WM, and decision making. Reversible MD dysfunction obtained by applying high-frequency stimulation caused significant deficits in the task. The authors concluded that the MD connects retrospective sensory with prospective action representations. On the whole, intracranial recordings suggest a role of the MD in directing cortical allocation of attention and thereby setting the stage for persistent cortical activity to occur by regulating prefrontal oscillations in a time-sensitive manner.

## The MD: An Enhancer of Frontal Lobe Function?

Recent human studies support the idea that the MD subdivisions are involved in multiple cognitive operations subserved by different areas of the frontal lobes. Intracranial recordings especially suggest that the temporal regulation of the interaction between the MD and the PFC is crucial to cognition, while similar evidence is not currently available for other brain regions like the perirhinal cortex. Still, the evidence appears inconclusive regarding the identification of a precise set of cognitive operations mapping onto the MD. Instead, it is possible that the MD plays a role in influencing the functions of multiple areas in the PFC and in other cortical regions.

We propose that the common ground of multiple lines of evidence from human studies points to a role of the MD in maintaining and temporally extending prefrontal activity patterns regardless of whether such activity represents rules [7], goals, memoranda, or subjective feelings of familiarity and recollection [83]. This MD influence on prefrontal activity patterns is likely critical when the PFC is required to perform tasks that require information to be online across a long delay, or for the management of cognitive interference, or during multitasking.

Influential views on the functions of the thalamus have emphasized its active gating properties with respect to stimuli directed from the periphery to the cortex, or from the cortex to other cortical areas via trans-thalamic routes 87, 88, 89, 90. The human PFC may be able to undertake many tasks without a functional MD, even WM tasks that, in rodents, are challenged by MD lesions. However, the evidence we reviewed suggests that the MD may actively enhance prefrontal excitability (i.e., increase the amplitude or duration of cortical activity) [91]. Active enhancing allows a more nuanced influence from the MD on cortical functioning compared with gating. Such a model is consistent with the idea that the effect of MD dysfunction may only become apparent with longer delays or demanding tasks that are temporally extended beyond the reach of WM (perhaps with a role in promoting prefrontal plasticity [56]). Nevertheless, some questions might remain unanswered by the ‘enhancer’ model. Since the PFC already hosts reverberating circuits, research on the human thalamus needs to investigate the specific contribution of MD-mediated cortical processing.

## The Trans-Thalamic Route: The MD as a Regulator

In humans, recurrent circuits within the PFC may be sufficient for short time intervals (e.g., within the span of WM), while the MD regulation of prefrontal oscillations may promote the temporal extension of PFC activity. A recent study [92] noted that the precision of WM representations degrades across long delays and further mechanisms may be needed to preserve it for longer retention intervals, mechanisms beyond the ‘enhancement’ of spiking activity. For example, the currently prevailing WM model includes a dedicated system to preserve multimodal memory representations across delays (episodic buffer) [93]. Besides its enhancing capacity, intracranial recordings show that the MD regulates cortical oscillations through signals directed from the MD to the PFC with a behavioral significance – even before stimulus onset and even unrelated to explicitly defined task goals [85]. The MD may thus be part of a network bridging past with future activity patterns across multiple cortical PFC regions [18]. This ‘connecting’ role may also explain why its function has been elusive so far: most of the operations are performed in the PFC. Notably, a regulating role of the MD does not necessarily imply that it drives cortical activity, but rather supports it during longer epochs of time. During this lapse of time, dynamic shifts between these regions may be required depending on their functional specializations, which might represent interfering processes that ultimately affect representation precision.

**Figure 2:**
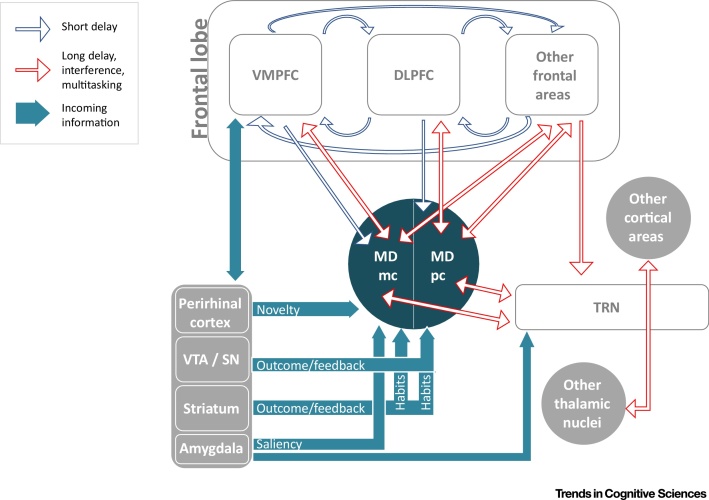
Key Figure: Regulation of the Prefrontal Cortex

A regulator differs from an enhancer because it undertakes integrative functions. While multiple frontal regions temporarily store persistent activation patterns in MD subdivisions by stimulating thalamic neuronal activity across the delay, new stimuli and changes to internal states will affect PFC activity patterns (Figure 2, Key Figure). At longer delays or because of multitasking and interference, this simultaneous activity may degrade the representations maintained by the PFC. In this scenario, the activity induced in the MD may be projected back and overrun degraded cortical reverberation patterns as well as cognitive interference. A critical aspect that makes the MD contribution different from corticocortical connections is the larger weight of thalamocortical synapses compared with cortico-cortical synapses 94, 95. By contrast, incoming information may affect the same system to turn to the novel (from the perirhinal cortex) or salient information (from amygdalar/mesencephalic inputs), effectively updating the cognitive state [13]. Such an updating function of the MD has been proposed in the framework of predictive coding, whereby the MD would represent a ‘Bayesian observer’ receiving as input *a priori* predictions from the cortex and projecting *a posteriori* predictions to the same or other cortical areas [96]. The Bayesian observer model is compatible with the regulatory role we propose, although our view emphasizes a threshold model whereby this activity becomes relevant (i) selectively in conditions entailing the degradation over time or due to interference of persistent activity patterns, and (ii) whether or not the information is updated, by antagonizing representation degradation. The fact that the MD, like the PFC, projects to multiple fields within the thalamic reticular nucleus (whereas other nuclei are connected to specific thalamic reticular fields [97]) also enables the MD to influence other thalamocortical networks. This circuit extends its influence across other regions of the brain, hence also suggesting a role for the MD in the spatial extension of the signal.

This model could explain why the role of the MD may be most relevant when the PFC is multitasking for long time intervals. These aspects of MD functions may be best investigated using carefully controlled interference [42] in studies affording high temporal resolution, for example, intracranial recordings (see Outstanding Questions). Overall, understanding the role of the MD in human cognition will likely require dedicated tests tapping into various cognitive functions rather than LTM alone, with a focus on temporal parameters of the task (Box 3).

## Concluding Remarks

Like Ulysses’ bed in the Odyssey, the thalamus is rooted in the center of the brain and this feature has constituted a formidable challenge for human neuroscience research. As new techniques allow us to peek inside the functions of specific thalamic nuclei, the MD is emerging from its purported function in supporting recognition and is, instead, beginning to command a higher profile in cognitive, behavioral, and clinical neuroscience.

We suggest reconsidering the focus on LTM that has characterized part of the literature in the past years. We argue that the MD role is more widely related to the maintenance and temporal extension of persistent activity in the frontal lobes. New studies should investigate thalamic nuclei separately with multimodal imaging assessments whenever possible (with special consideration of functional connectivity approaches), and include lesion quantification, possibly accounting for bias in the neuroimaging estimates. Only specifically designed neuropsychological tests – as opposed to routine assessments – in tandem with state-of-the-art fMRI sequences and data analysis performed in a consortium framework will achieve sample sizes suitable to approach the conundrum on the function of the human MD.

Outstanding Questions How to push for more precision and accuracy in estimates of the MRI signal (volumetric, diffusion, or blood oxygen level dependent) from the medial thalamus to obtain measurements that are truly specific to alterations in the medial thalamus and not artifactual because of noise, lesions, or a mixture of signal from neighboring thalamic nuclei? This technical improvement would be important to understand alterations in pathological conditions like SCZ and AUD.

How can the efficiency of MD–PFC interactions be quantified in humans for the development of neuroimaging targets for therapy in clinical conditions characterized by MD dysfunction?

What is the cognitive advantage of the trans-thalamic route of corticocortical communication? Are specific triangular circuits involving the MD mediating specific cognitive operations?

How does MD dysfunction affect temporal aspects of performance, as assessed, for example, by implementing variable response deadlines, interference at variable times along the task, and variable response delays over several minutes?

Is the MD specifically related to multitasking and interference management?

What is the cognitive complaint of patients with MD lesions in the long run?

What are the brain-wide alterations in gray and white matter following MD lesions?

What are the effects of unilateral versus bilateral MD lesions on cognition? To what extent do the projections from subdivisions of the MD nucleus overlap in the frontal lobes?

## Glossary

**Attention control**: attention-related tasks in real life require ignoring a variety of distractions and inhibiting attention shifts to irrelevant activities. Attention control consists of the top–down allocation of attentional resources to perform a variety of cognitive tasks.
**Executive functions**: the capacity of the brain to formulate goals, plan, and carry out plans effectively [98].
**Long-term memory**: the ability to remember learned material for time spans from minutes to a lifetime, including a variety of memory systems (e.g., episodic, semantic, procedural), which share the storage of memory representations for a time exceeding the persistence of the information in the stream of consciousness.
**Mediodorsal thalamic nucleus**: nucleus of the dorsal thalamus, located in the anteroposterior axis below the anterior nucleus, at the midline, medially to the internal medullary lamina. It has at least two different subdivisions: the medial magnocellular MD and the central parvocellular MD. A third subdivision, the lateral MD, is included by some authors among the intralaminar nuclei [94]. These subdivisions each receive afferents originating from different parts of the brainstem, midbrain, basal ganglia, prefrontal cortex, and limbic system. Different MD subdivisions project to different frontal areas, and anterior cingulate and insular cortex. The paramedian and tuberothalamic arteries support perfusion on its medial and rostrolateral borders, respectively.
**MRI, structural and functional (fMRI)**: Structural imaging provides static anatomical information translating the local molecular differences into different shades of gray to outline the shape and size of the brain regions. An MRI scanner delivers a specific radiofrequency that excites hydrogen atoms, which return some of this energy in the form of a characteristic nuclear magnetic resonance signal. Functional imaging supplies dynamic physiological information indirectly related to metabolic changes in the neural tissue, including blood oxygen level-dependent contrast, perfusion (whether by endogenous or exogenous contrast), blood flow, and cerebrospinal fluid pulsation.
**Prefrontal cortex**: the most rostral part of the frontal lobe, which coordinates a wide range of neural processes and includes interconnected neocortical areas that send and receive projections from virtually all cortical sensory systems, motor systems, and many subcortical structures.
**Thalamus**: a complex of 50–60 nuclei, located in the diencephalon, spanning both hemispheres. The rostro-caudal dimension of the human thalamus is about 30 mm, its height about 20 mm, and its width about 20 mm, with about 10 million thalamic neurons in each hemisphere.
**Working memory**: a limited capacity system with load, interference control as well as updating functions, which temporarily maintains and stores information. An influential model of working memory includes a central executive (in charge of executive functions) and three slave systems for passive short-term storage of verbal, visuospatial, or multimodal (episodic buffer) information [93]. It is believed to support human thought processes by providing an interface between perception, long-term memory, and action.
